# Connecting the Dots: Telomere Shortening and Rheumatic Diseases

**DOI:** 10.3390/biom14101261

**Published:** 2024-10-06

**Authors:** Fang Han, Farooq Riaz, Jincheng Pu, Ronglin Gao, Lufei Yang, Yanqing Wang, Jiamin Song, Yuanyuan Liang, Zhenzhen Wu, Chunrui Li, Jianping Tang, Xianghuai Xu, Xuan Wang

**Affiliations:** 1 Department of Rheumatology and Immunology, Tongji Hospital, School of Medicine, Tongji University, No. 389 Xincun Road, Shanghai 200065, China; hanfang2022@tongji.edu.cn (F.H.); 1910829@tongji.edu.cn (J.P.); 2033349@tongji.edu.cn (R.G.); y_l_f@tongji.edu.cn (L.Y.); 2305553@tongji.edu.cn (Y.W.); 2480126@tongji.edu.cn (J.S.); 1632202@alumni.tongji.edu.cn (Y.L.); 1733041@alumni.tongji.edu.cn (Z.W.); 2332261@tongji.edu.cn (C.L.); tangjp6512@tongji.edu.cn (J.T.); 2 Faculty of Pharmaceutical Sciences, Shenzhen University of Advanced Technology, Shenzhen 518000, China; farooq.riaz@siat.ac.cn; 3 Center for Cancer Immunology, Shenzhen Institute of Advanced Technology (SIAT), Chinese Academy of Sciences (CAS), 1068 Xueyuan Avenue, Shenzhen 518055, China; 4 Department of Pulmonary and Critical Care Medicine, Tongji Hospital, School of Medicine, Tongji University, No. 389 Xincun Road, Shanghai 200065, China; 05849@tongji.edu.cn

**Keywords:** telomere, telomere shortening, telomerase, telomerase reverse transcriptase, rheumatic diseases, autoimmunity

## Abstract

Telomeres, repetitive sequences located at the extremities of chromosomes, play a pivotal role in sustaining chromosomal stability. Telomerase is a complex enzyme that can elongate telomeres by appending telomeric repeats to chromosome ends and acts as a critical factor in telomere dynamics. The gradual shortening of telomeres over time is a hallmark of cellular senescence and cellular death. Notably, telomere shortening appears to result from the complex interplay of two primary mechanisms: telomere shelterin complexes and telomerase activity. The intricate interplay of genetic, environmental, and lifestyle influences can perturb telomere replication, incite oxidative stress damage, and modulate telomerase activity, collectively resulting in shifts in telomere length. This age-related process of telomere shortening plays a considerable role in various chronic inflammatory and oxidative stress conditions, including cancer, cardiovascular disease, and rheumatic disease. Existing evidence has shown that abnormal telomere shortening or telomerase activity abnormalities are present in the pathophysiological processes of most rheumatic diseases, including different disease stages and cell types. The impact of telomere shortening on rheumatic diseases is multifaceted. This review summarizes the current understanding of the link between telomere length and rheumatic diseases in clinical patients and examines probable telomere shortening in peripheral blood mononuclear cells and histiocytes. Therefore, understanding the intricate interaction between telomere shortening and various rheumatic diseases will help in designing personalized treatment and control measures for rheumatic disease.

## 1. Introduction

Telomeres are repetitive sequences and nucleoproteins that serve as protective caps at the ends of eukaryotic chromosomes [1,2]. They include the greatly conserved hexameric tandem repeats DNA sequence motif (TTAGGG) [3]. Telomeres are essential in maintaining the stability and integrity of the genome as well as assisting in evolution [4,5,6]. It has been established that telomere length (TL) is a crucial regulator of cell proliferation and apoptosis [7]. Sufficiently longer telomeres serve as a protective barrier, effectively inhibiting the initiation of the DNA damage response [8]. Meanwhile, chromosomal capping is compromised by telomere shortening, which may limit cell proliferation and increase cell apoptosis [9]. The shelterin complex, which is made up of six proteins, is responsible for maintaining the length of telomeres. These proteins are as follows: POT1, RAP1, TRF1, TRF2, TRF1- and TRF2-interacting nuclear protein 2 (TIN2), telomeric repeat binding factor 1 and 2 (TRF1, TRF2), and POT1-TIN2 organizing protein (TPP1) [10]. Telomere DNA repeats bind to shelterin, inhibit DNA repair, and distinguish between healthy chromosomal ends and damaged breaks in order to maintain telomere length. To lengthen telomeres, telomerase appends repetitive sequences to their ends through its two subunits: telomerase RNA component (TERC) and telomerase reverse transcriptase (TERT) [11,12,13]. Individuals susceptible to complicated conditions may have aberrant telomerase function or telomere shortening [14].

Researchers have revealed that TL is linked with various cellular, biological, and pathological processes. Short telomeres may serve as a hallmark of cellular aging and oxidative stress in multiple diseases, including cancer [15], cardiovascular disease [16,17], and death [18,19]. Individuals suffering from these diseases tend to have relatively short telomeres compared to young/healthy individuals, suggesting that the shortening of telomeres may crucially impact the development and progression of these conditions. Meanwhile, it has also been understood that telomere shortening participates in the development of multiple diseases through cellular apoptosis or senescence and activating DNA damage responses that can trigger inflammation and oxidative stress [20,21,22]. These biological activities can result in further telomere shortening and the accumulation of genetic mutations [23,24], eventually leading to age-related disorders [23].

Rheumatic disorders cause inflammation and tissue damage. Numerous studies show that persons with rheumatic illnesses have shorter telomeres than healthy people, indicating that telomere shortening may promote disease development. Due to its role in immune cell abnormalities, telomere dysfunction has become a promising treatment for rheumatic disorders. Many studies have found that telomere shortening, along with reduced immune cell activity in rheumatic diseases, causes organ damage and progression to primary Sjögren’s syndrome (pSS), systemic lupus erythematosus (SLE), rheumatoid arthritis (RA), osteoarthritis (OA), and systemic sclerosis (SSc) [25]. Furthermore, there is a strong correlation between telomere shortening and the onset of interstitial lung disease (ILD) [26,27]. Gamal and Svyryd et al. found that chromosomal telomere shortening in RA patients was positively connected with disease activity and linked to a higher chance of developing RA [28,29]. Telomere disruption is associated with an increased chance of developing SLE, according to research conducted by Tsai and Gao et al. They also concluded that SLE patients may have telomere dysfunction due to early telomere erosion, which can result in immunosenescence, decreased tissue regeneration, and endothelial cell damage [30,31]. Numerous studies have revealed telomere shortening and malfunction in different tissue cells of pSS patients. As Onuora et al. found that gout patients had shorter TLs, which may be related to the increased leukocyte turnover brought on by inflammation, inflammation is a crucial component in this process [32].

Here, we demonstrate the current understanding of the relationship between telomere dynamics and rheumatic disorders, which may lead to the development of customized therapies and diagnostic indicators. Briefly, our study discusses the factors that influence the length of telomeres and the potential mechanisms of telomere shortening. Likewise, based on the literature on telomerase length in rheumatic patients, we also focus on introducing the diagnostic markers that could result in the early and precise detection of diseases, thereby revolutionizing clinical management. In conclusion, this review paper is critical in determining the future direction of telomere dynamics in rheumatic diseases.

### Structure and Function of Telomere Complex

Two primary components make up the structure of the telomere complex. These include specific DNA repeat sequences and a set of proteins known as “shelterin” or the telomere shelterin complex. Griffith et al. first described the looped structure of telomeres, known as the “T-loop”, and discussed the role of the shelterin complex in forming and maintaining this structure [33], whereas Lange et al. conducted an in-depth discussion on how the shelterin complex interacts with telomere DNA and protects telomeres from damage [10]. Short repetitive sequences, known as G- and T-rich tandem repeats in most eukaryotes, make up telomeric DNA. These repetitions serve as a protective cap structure that prevents the cellular DNA repair mechanism from mistaking the ends of the chromosomes for breaks. They do not encode any proteins [34].

A particular set of proteins, known as the shelterin complex, work directly with the DNA of telomeres to create a protective structure. Six proteins make up the shelterin complex: TRF1, TRF2, RAP1, TIN2, TPP1, and POT1. While TRF1 and TRF2 directly connect to the double-stranded DNA of telomeres, POT1 binds to the single-stranded 3′ G-rich overhang. Through these bindings, shelterin stabilizes the telomere structure, preventing DNA damage response mechanisms from misinterpreting telomeres as breaks in DNA. The shelterin complex can shield telomeres in a variety of ways. For instance, TRF2 and Rap1 are involved in the formation and maintenance of the telomere loop, or T-loop. This structure helps to prevent telomere ends from being mistaken for DNA damage by the mechanisms involved in cellular DNA repair and recombination. As a result, there is a reduced likelihood of unnecessary repairs and chromosomal instability developing. TRF2 also contributes to the suppression of the ABM signaling pathway, which is the body’s response to breaks in DNA strands. Both the ABM signaling channel and the ABM signaling pathway are suppressed in part by TRF2. Moreover, telomerase’s binding to telomere DNA is improved by the complex produced by TPP1 and POT1, which facilitates telomere lengthening. Additionally, TIN2 connects TRF1 and TRF2 to additional proteins [10].

## 2. Factors Affecting Telomere Length

Genetic and environmental factors considerably impact telomere length [35,36]. Numerous exogenous factors, such as toxins, poisons, and pollution, impact telomere length and result in harmful effects on cells [37,38,39,40,41]. The research conducted by Ikeda and colleagues revealed that UV radiation exposure causes DNA damage, which, therefore, affects telomere length [42]. Telomeres can shorten as a result of unhealthy habits, including smoking, binge drinking, and poor eating [43,44,45]. In addition, telomere shortening has been linked to stress, inactivity, and inadequate sleep. Due to the shortened telomeres caused by chronic psychological stress, age-related illnesses are more likely to occur [46]. Meanwhile, people suffering from viral infections, particularly those with multiple viral infections, have noticeably lower telomere length compared to unaffected people [47,48]. A recent study also concluded that a small portion of telomeric DNA is lost during cell division due to the end-replication problem, where DNA polymerases cannot fully replicate the ends of linear chromosomes. This gradual loss leads to progressively shorter telomeres over successive divisions [49].

Dietary habits, such as a diet rich in vital minerals, vitamins, and antioxidants, as well as the diet being well balanced may help preserve telomere length and reduce oxidative stress [50,51,52]. Conversely, processed foods, elevated blood sugar, and extra lipids can also shorten telomeres and harm cells [53]. Apart from environmental considerations, genetic factors are also essential for maintaining the length of telomeres. This includes the regulation of the telomerase activity rate during the process of telomere degradation [54]. Telomerase-related genes (TERC, TERT) [55] and telomere shelterin complex-related genes (TRF1, TRF2, TIN2, POT1, TPP1, and RAP1) [10,56] are known to regulate TL and stability. Recent studies have revealed that the telomerase component hTERT shortens excessively long telomeres while elongating short ones. This regulatory function is crucial for maintaining telomere length homeostasis and involves interactions with shelterin proteins, such as TPP1, which help recruit telomerase to telomeres [57]. Cellular nucleotide metabolism, particularly thymidine, regulates genomic integrity and telomere length [58,59]. Additionally, alterations in hormone levels, such as alterations in estrogen levels after menopause in women or androgen levels in males, can affect TL [60]. Studies have demonstrated that certain reproductive hormones can influence the functioning of telomeres. Estrogen possesses anti-inflammatory and antioxidant characteristics and can stimulate telomerase expression. Testosterone, the main male sex hormone, is believed to protect against the risk of developing multiple sclerosis [60,61]. Notably, the ageing rate is different in men and women and is highly dependent on sex hormones [62]. These findings broaden our comprehension of how various environmental factors, probably under the influence of genetic factors, can uniquely impact the diverse DNA synthesis mechanisms and TL.

Moreover, telomere dysfunction can lead to various biological and pathological consequences. This dysfunction typically occurs when telomeres shorten to a critical length and are unable to protect chromosome ends effectively. Such shortening can induce cellular senescence, apoptosis, or carcinogenesis [63]. Telomere dysfunction not only leads to direct genomic alterations but also contributes to broader chromosomal instability. This includes an increased risk of mutations, unbalanced translocations, and deletions, hallmark features in conditions like myeloid neoplasia. The instability is exacerbated by defects in telomere maintenance mechanisms, which can influence disease progression and response to therapy [64]. The dysfunctional telomeres can directly lead to telomere–telomere fusions, creating complex chromosomal rearrangements and aneuploidy [65]. By deepening our understanding of how telomere shortening contributes to cellular and genetic instability, we can better devise strategies to mitigate these effects.

## 3. Possible Mechanisms of Telomere Shortening in Inflammation

**Figure 1:**
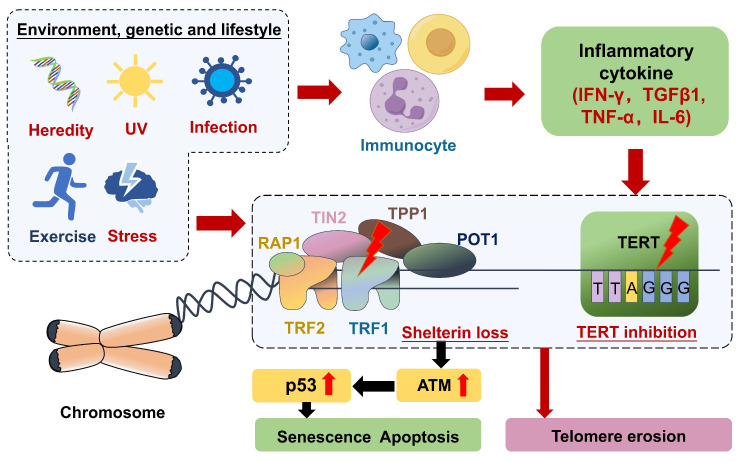
Two possible mechanisms by which telomere shortening contributes to the development of rheumatic diseases. Telomere erosion can be brought on by one of two pathways under the impact of genetic, environmental, and lifestyle factors. One mechanism is that ROS generated by chronic inflammation or oxidative stress cause irreversible damage to telomere DNA, and at this point, telomere shelterin loses its protective effect on telomere DNA. DNA damage or the absence of shelterin leads to an increase in the activity of ATM, which, in turn, activates the p53 gene, leading to cellular senescence or apoptosis. Another mechanism is to cause an imbalance in the body’s immune system, whereby inflammatory factors released by immune cells and associated antibodies act on telomerase, leading to telomere dysfunction, thus inhibiting telomere lengthening. UV: ultraviolet; IFN-γ: interferon-γ; TGF1β: transforming growth factor β1; TNF-α: tumor necrosis factor-α; IL-6: interleukin- 6; TERT: telomerase reverse transcriptase; ATM: ataxia telangiectasia-mutated. Shelterin is composed of six proteins (POT1, TPP1, TRF1, TRF2, TIN2, and RAP1).

Immune cell activation and environmental, genetic, and lifestyle factors collectively contribute to abnormal telomere function in patients with rheumatic diseases. Possible mechanisms of telomere shortening in the development of rheumatic diseases can be broadly categorized into two main categories: telomere replication problems and oxidative stress damage (Figure 1). Telomere replication problems occur when DNA polymerase fails to replicate the terminal of linear DNA, resulting in the loss of a small telomere segment after each replication, known as the “telomere end problem” [66]. Additionally, factors such as the cell’s replicative history and its ability to maintain telomere length can influence this process, leading to variability among different cell types [56]. As telomeres shorten, T cells—especially CD8^+^ and CD4^+^ T cells—experience reduced proliferative capacity. This limits their ability to replicate in response to infections or other immune challenges, leading to a weakened immune response. Shorter telomeres in these cells are associated with cellular senescence, which means they are less responsive [67]. In autoimmune diseases, both naive and memory T cells exhibit premature aging due to telomere shortening. Naive CD4^+^ T cells show reduced telomerase activity upon stimulation, leading to increased apoptosis and limited clonal expansion, contributing to immune dysregulation [68]. Concurrently, oxidative stress damage is due to telomere sequences being rich in guanine and susceptible to damage by reactive oxygen species (ROS), resulting in telomere breakage or shortening [20,21].

Cells are likely predisposed to apoptosis and senescence due to damage to telomeric DNA caused by ROS generated by defective mitochondria or signaling pathways [69,70]. The imbalance between the antioxidant defense system and the production of ROS leads to oxidative stress. Chronic oxidative stress has been linked to cellular damage and, in the presence of various external and rheumatic factors, the structure of DNA is damaged or mutated, which may cause functional changes in shelterin complexes and telomerase. Furthermore, metabolic shifts associated with inflammation—such as alterations in glucose metabolism and mitochondrial dysfunction—can exacerbate ROS production, further contributing to telomere attrition [71]. DNA damage or loss of shelterin leads to increased ataxia telangiectasia-mutated (ATM) activity, which activates the p53 gene and causes cellular senescence or apoptosis [46,72,73,74].

In rheumatic diseases, oxidative stress can be caused by ROS produced by activated immune cells and/or inflammation-induced oxidative damage to the joints. Inflammatory cytokines, such as interferon-gamma (IFN-γ) and tumor growth factor beta-1 (TGFβ1) [75], can directly affect TL by inhibiting telomerase activity and promoting telomere attrition. The interplay between these cytokines and immune cell activation forms a feedback loop that accelerates telomere shortening, leading to a decline in immune function. The release of pro-inflammatory cytokines activates immune cells. Among these immune cells, B cells are thought to drive the production of associated antibodies that affect telomerase activity [76]. In addition, inflammation can increase oxidative stress, which further contributes to telomere shortening [77]. As a result, their ability to respond to antigens and their overall functioning are changed. This alteration is more prevalent in the elderly and is linked to a weakened immune system, particularly in the face of recent illnesses [78].

Aging may have an effect on dendritic cell activity, which is crucial for both triggering and controlling immunological responses. With shorter telomeres, dendritic cells may exhibit impaired maturation and reduced capacity for antigen presentation, diminishing their role in activating T cells and eliciting adaptive immune responses [79]. Telomere shortening may cause dendritic cells to react differently to and display antigens. This might result in a reduction in immunological surveillance, heightened vulnerability to infections, and the advancement of cancerous growths [79]. Recent research indicates that when macrophages age, their telomeres shorten. This is connected to a decline in their capacity to operate and a decline in STAT5a phosphorylation, which is essential for their immune response mechanisms [80]. Furthermore, “inflammaging”—a persistent, mild inflammation seen in the elderly—is brought on by alterations in macrophages brought about by aging. Tissue inflammation is made worse by a change in macrophage activity toward pro-inflammatory characteristics, which is the hallmark of this condition. Aged macrophages have diminished anti-inflammatory capabilities, which makes it harder for them to preserve tissue homeostasis, primarily due to instabilities in their phagocytosis and antigen presentation mechanisms [81]. These findings emphasize that preserving telomere integrity is critical for a higher activity of macrophages. Furthermore, targeted treatment to preserve telomere length could reduce immunological dysfunction linked to aging and persistent inflammation.

Natural shortening of TL occurs in individuals as they become older. This telomere shortening may lead to decreased cellular function and increased risk of infections, cancers, and inflammatory diseases [18]. Features of immune senescence include hypothyroidism, expansion of effector T cells, telomere shortening, and overproduction of cytokines [31]. The most studied rheumatic disease concerning immune aging is RA. T cell senescence plays a crucial role in RA. The thymus is the principal site of T cell development and maturation. As humans age, there is shrinkage of the thymus, which decreases the production of naive T cells, leading to reduced T cell diversity and a predominance of memory T cells [82]. The thymus function in RA patients is diminished earlier and more severely than normal due to environmental or genetic factors [31,83]. Hypothyroidism, in turn, affects T cell tolerance to autoantigens and central regulation [84]. Senescence-associated secretory phenotype (SASP) in senescent T cells helps them secrete large amounts of pro- and anti-inflammatory cytokines such as interleukin (IL)-10, IL-6, IL-1β, and tumor necrosis factor (TNF-α) [31,85]. These cytokines can influence the differentiation, activation, polarization, and migration of T cells to affect other immune cells and target organs, leading to systemic inflammatory responses and tissue damage.

Genetic factors have a distressing role in telomere shortening and developing rheumatic diseases. Studies have proven the association of telomere shortening with RA, primarily due to telomere maintenance-related genes, such as TERC and TERT [86]. From this perspective, genetic susceptibility to rheumatic diseases may also influence TL and telomere attrition rates [87]. Although these factors have the potential to influence TL, the correlation between TL and illness is intricate and not yet comprehensively understood. Additional investigation is required to ascertain the methods for preserving robust telomeres and mitigating telomere diminishment. Hence, the potential processes responsible for telomere shortening in the advancement of rheumatic diseases encompass an intricate interplay among oxidative stress, inflammation, and genetics. Comprehending these mechanisms is crucial for the development of precise treatments to hinder the process of telomere shortening and to limit the occurrence of inflammatory and rheumatic disorders. It is worth mentioning that TL might differ among various tissues and cell types within the same individual. As an illustration, blood cells often possess shorter telomeres compared to skin cells, and the length of telomeres might differ according to the phase of cell division

## 4. Telomere Erosion in Rheumatic Disease

**Figure 2:**
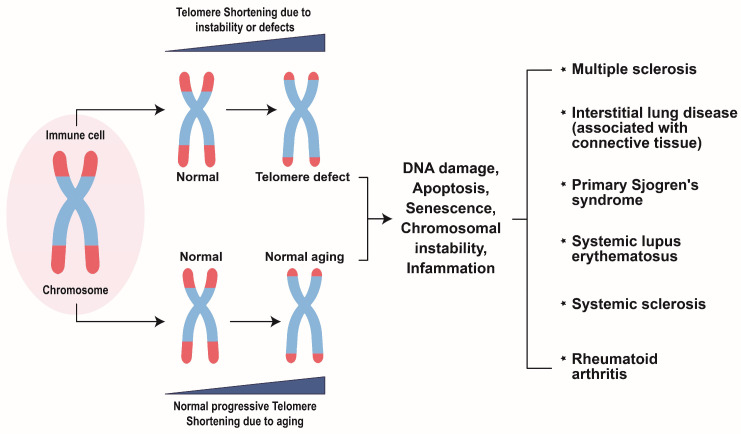
Schematic representation of the progression of autoimmune diseases due to telomere shortening. In normal individuals, cells undergoing continuous division develop shorter telomeres. A successive shortening of telomeres results in dysfunctional telomeres. This induces cell apoptosis, cell senescence, DNA damage, and chromosomal instability and may lead to inflammation and ultimately progress to the onset of autoimmune diseases. Meanwhile, genetic disorders or telomere defects may also lead to DNA damage and cellular dysfunctions, leading to the progression of autoimmune diseases.

The specific problems encountered by semi-conservative DNA replication are caused by the arrangement of telomeres [56,88]. Due to the limitations in replicating chromosome ends, often called the “end replication problem”, telomeres gradually shorten over successive cell divisions [89,90]. When telomeres reach a critical short length, a checkpoint response is triggered, potentially resulting in cell senescence or apoptosis. In cases where these checkpoints malfunction, telomeres continue to shorten and lose their protective features, entering a state known as “crisis” [91]. The crisis is regarded as genome instability and carries the risk of cell death or cellular transformation. A recent study elucidated that crisis-dependent cell death and cellular transformation are dependent on the activation of the innate immune response and IFN signaling, mainly due to the interaction of telomeric-repeat-containing RNA (TERRA) and Z-DNA binding protein 1 (ZBP1) [92,93]. These inflammatory IFN signaling and innate immune responses are associated with the progression of autoimmune and rheumatoid diseases (Figure 2) [94,95,96]. Below, we discuss the association between telomere shortening and dysfunction in various rheumatoid diseases.

### 4.1. Rheumatoid Arthritis

RA is an inflammatory autoimmune disease that mainly encompasses synovial inflammation and ultimately progresses to cartilage damage and destruction of joint infrastructure [97]. It has been illustrated that immunological changes during the progression of RA are somehow dependent on dysfunctional telomeres and the accumulation of autoreactive and senescence T cells [31,98,99]. In a 2007 study, Steer et al. found that a reduced the length of the terminal restriction fragment (TRF) in patients with RA was not independent of disease duration and markers of disease severity [100]. Ormseth et al. also found no striking difference in TL in RA patients compared to healthy individuals. However, more studies have found the opposite result [101]. Gamal et al. found that TL was considerably shorter in patients with RA than in normal individuals and that oxidative stress markers increased as disease activity increased [28]. Through cross-sectional and longitudinal studies, Svyryd et al. found that RA patients had different degrees of telomere shortening at different disease stages [29]. In Salmon’s study, telomere loss in peripheral T cells was increased in RA patients [102]. This may be a property of HLA DR4 expression in all blood cells and represents a fresh viewpoint on the mechanism underlying HLA illness correlations. Meanwhile, Yudoh et al. demonstrated that synovial infiltrating lymphocytes and peripheral blood leukocytes (PBLs) of RA patients exhibit higher telomerase activity [103]. This higher telomerase activity in lymphocytes may help in understanding the inflammation, disease progression, and synovial proliferation in RA. As mentioned by Fujii et al., in their study, due to the inadequate induction of the telomerase component hTERT, RA derived naive CD4^+^ T lymphocytes were poor in elevating telomerase activity after stimulation [68]. These basic and clinical studies [99,104,105,106,107] provide additional evidence for the link between telomere shortening and RA, including genetic variants, sheltering complexes, and alterations in telomerase activity. The clinical relevance of telomere shortening and RA is described in Table 1.

### 4.2. Systemic Lupus Erythematosus

The rapid aging of the immune system, marked by shortened telomeres or immune senescence, is linked to systemic lupus erythematosus (SLE), as telomere shortening contributes to the initiation and advancement of SLE [145,146]. Earlier, in an observational study, Honda et al. found that peripheral blood mononuclear cells (PBMCs) from SLE patients exhibited shorter telomeres and fewer mitotic cycles compared to controls [123]. In two successive published studies, Kurosaka et al. found a significant correlation between the telomerase activity of PBMCs and changes in the systemic lupus erythematosus disease activity index (SLEDAI), particularly in B lymphocytes [119,122]. Subsequent studies have also demonstrated higher telomerase activity and shorter TL in SLE patients [114,115,116,118,120,121], but the degree of increased telomerase activity does not yet offset the telomere shortening.

Using two independent samples of European and Chinese ancestry, a recent study by Wang et al. utilized a two-sample Mendelian randomization approach. It investigated the impact of TL shortening on SLE. Contrary to other investigations, this study yielded that longer TL was significantly linked with an enhanced risk of SLE [147]. The results of this Mendelian analysis were the opposite of previous observational studies, and perhaps the case controls included in previous observational studies did not perform well in excluding the effects of other confounding factors, such as medication use. On the flip side, there may also be some limitations to the study by Wang et al., as their team only selected blood leukocyte TL measurements and did not take into account differences in different tissue sources. However, they did take into account differences by race. This would seem to make further research even more necessary. The conclusive pattern of telomere shortening in various SLE studies is illustrated in Table 1.

### 4.3. Primary Sjögren’s Syndrome

Previous studies have demonstrated varying degrees of telomere erosion in salivary, parotid, and PBMC DNA from pSS patients [126,127]. Noll et al. examined the level of relevant genes by quantitative polymerase chain reaction (qPCR). They found that not only were salivary DNA telomeres significantly shorter, but also, when compared to non-pSS sicca patients, labial salivary gland (LSG) tissues in pSS patients showed elevated levels of ATM mRNA and were closely linked with LEF1, TPP1, and POT1 [125]. In addition, overexpression of multiple inflammatory cytokines in salivary glands of pSS patients may affect the maintenance of TL and is associated with increased activation of seeded lymphocytes, dendritic cells, and release of cytokines. TGF-β1 and IFN-γ may influence TERT expression, thus affecting TL. Telomere erosion in pSS glandular vesicle cells is a critical factor in stimulating cell senescence, which involves ROS-associated DNA damage [124]. Table 1 summarizes the trend of telomere shortening observed in pSS. Distressingly, numerous studies with limited sample sizes and inadequate controls have precluded the definitive establishment of causality or the precise nature of the interplay between pSS and TL. Further research is imperative to comprehensively grasp the connection between TL and pSS and explore the viability of telomere shortening as a therapeutic target for pSS treatment.

### 4.4. Systemic Sclerosis

In patients with systemic sclerosis, various previous investigations have focused on the analysis of telomere length in PBL, and less similar results have been obtained by different telomere measurements. SSc patients had shorter TLs compared to healthy controls [134], and SSc patients with combined ILD appeared to have even shorter TLs [128,129,131]. In a study, Tarhan et al. found minimal hTERT activity in SSc patients, even lower than in healthy controls [132]. Similarly, Adler et al. found antibodies against telomerase and shelterin, and TERF1 antibodies were present in 40/442 (9%) of scleroderma patients [130]. Notably, in a study published in 2008, MacIntyre et al. unexpectedly found a longer mean TL for limited systemic sclerosis (LcSSc) by regression analysis, which was only significant in patients over 50 years of age [133]. Combined with later studies related to telomerase activity and telomerase antibodies, it is speculated that this result may have been obtained because the clinical subgroup of LcSSc chosen for that study and the Southern blotting method used was inevitably influenced by the sequence of the parachromosomal region [133]. Another possibility is that it is perhaps related to the severity of the disease, where at a certain period of the disease, relevant antigens or antibodies are produced in a short period, rapidly exacerbating inflammation. Considering the association of autoantigens with SSc, it was stipulated that telomeres and/or telomerase may act as autoantigens in SSc [148]. Meanwhile, in SSc biopsies, a connection was observed between mesenchymal cells exhibiting telomere-associated foci and the presence of CD38^+^ cells, primarily associated with cell senescence. This suggests that elevated CD38 levels could promote cellular senescence in SSc [149,150,151]. Table 1 provides insights into the clinical significance of telomere shortening in SSc.

### 4.5. Other Rheumatic Diseases

Most current studies discussing the association of TL and osteoarthritis patients have focused on PBL samples, and the conclusions are similar. These studies show that OA patients with shorter TL and short telomeres are a risk predictor for OA [135,136,137]. Meanwhile, Poonpet et al. found that plasma angiogenic cytokines, such as G-CSF, VEGF, and HGF, negatively correlate with the relative TL of leukocytes [138]. Only one study suggests that a dysfunctional telomere may aggravate small vessel vasculitis (SVV) through an aging-dependent mechanism [152]. It also raises the question of DNA damage, which could be a common problem for telomere shortening in patients with rheumatic diseases [152]. Short telomeres have also been found in rheumatic diseases such as spondyloarthritis [141] and gout [143]. By analyzing telomere length in PBMCs of patients with rheumatic diseases, Tamayo et al. found that not only did telomere length vary across diseases but also the degree of telomere shortening [109,142]. The diseases described above are just examples of rheumatic diseases that probably encompass premature aging mechanisms (Table 1). Nevertheless, investigations into other rheumatic diseases and their associated telomere length assessments are rare. Consequently, further research is indispensable to substantiate these findings.

### 4.6. Interstitial Lung Disease Associated with Connective Tissue Disease

Interstitial lung disease (ILD) represents a prevalent and clinically consequential feature of various connective tissue diseases (CTDs). ILD can manifest across a broad spectrum of CTD types, encompassing conditions such as RA, polymyositis or dermatomyositis, scleroderma, SLE, pSS, and mixed connective tissue disease. Several studies have confirmed the presence of shortened telomeres or reduced activity of immune cells in rheumatic diseases and are closely related to the development of interstitial lung disease [104,129,153,154,155]. It was investigated whether TL is linked with the progression of interstitial lung diseases [26]. A report by Armanios et al. concludes that there were TERC and TERT mutations in 8% of probands with familial idiopathic pulmonary fibrosis [86]. In a related study, Tsakiri found that about 15% of patients with familial interstitial pneumonia (FIP) had mutations in the TERT or telomerase RNA component (TERC) gene that reduce enzyme activity, leading to telomere shortening [156]. In addition, a study by the American Legion this year confirmed that patients with RA-ILD had significantly shorter telomere levels than those with RA [104]. This is similar to the above findings in SSc patients, where telomere shortening was more pronounced in patients with rheumatic disease combined with ILD. Therefore, we urge that a telomere length assay could be a key indicator to assess CTD-ILD occurrence.

## 5. Conclusions and Perspectives

It has been speculated that telomeres are associated with the development and progression of an array of diseases, and current investigations focus on potential therapies targeting telomers. In conditions involving inflammation, autoimmune, or rheumatoid diseases, the objective is to raise or preserve TL, but in cancer, the objective is to reduce the tumor cell proliferative potential. As a result, telomerase, TERT, TERC, and telomere-associated proteins are now therapeutic targets [157,158]. Telomerase activators like 08AGTLF, TA-65, MA (maslinic acid), and OA (oleanolic acid) have demonstrated potential in terms of its anti-ageing properties and managing inflammatory diseases, including rheumatoid arthritis. These compounds can stimulate telomerase activity, which may help maintain telomere length and mitigate cellular aging and inflammation, potentially alleviating symptoms associated with chronic inflammatory diseases [39]. In this review, we have consolidated insights into rheumatoid-related conditions, shedding light on the frequently overlooked role of telomere dysfunction, whether through telomere shortening or telomere DNA damage, as an underlying cause. Recognizing telomere dysfunction as a contributory factor opens avenues for potential therapeutic interventions to treat rheumatic diseases. Ongoing preclinical studies in this realm involve strategies to mitigate telomere shortening and to counteract telomere DNA damage response (tDDR) activation, ultimately addressing cellular senescence [159,160]. Some researchers are investigating telomere-lengthening therapies to slow or reverse disease progression in patients with rheumatic diseases. These therapies aim to lengthen the telomeres of affected cells, potentially reducing cellular senescence and oxidative stress and improving cellular health. While more research is needed to fully understand the role of telomere shortening in rheumatic diseases and its potential as a diagnostic and therapeutic target, current findings suggest that the use of telomere length measurements and telomere-based therapies in the management of rheumatic diseases has excellent potential.

We reviewed that the DNA damage response, telomerase activity, and the presence of relevant anti-telomerase antibodies, together with several congenital genetic factors, all affect telomere length to a greater or lesser extent. However, how these factors exert their roles in modulating the course of the disease has not been studied, and this needs further investigation. As the potential role of telomere shortening in the diagnosis and prognosis of rheumatic diseases is increasingly investigated, shorter telomeres may be associated with more severe disease progression and poorer outcomes. Surprisingly, most human telomere and rheumatoid disease studies have assessed TL or telomerase activity in blood samples and pro-inflammatory cells. This opens the door for future clinical studies to comprehensively understand the impact of telomere shortening in immunosuppressive and anti-inflammatory cells, such as regulatory T cells. Meanwhile, regarding diagnostic value, telomere length measurement can probably be used as a diagnostic marker to differentiate rheumatic diseases from other related disorders.

Although there have been seemingly conflicting views on the association of telomere length and rheumatic disease, given that telomeres shorten with age, it is unclear whether short telomeres cause disease or disease causes short telomeres. It is possible that both mechanisms are at play and that the link between telomere length and rheumatic disease may be bidirectional, with a dominant party between the two.

However, this review is also limited in that our study focused only on short-lived immune cells, and this information may, in principle, apply to all autoimmune diseases and not only to rheumatic diseases. It is also essential to consider the limitations of the current research on telomere length and rheumatic disease. Many studies use small and poorly controlled sample sizes, and the methods and cellular sources used to measure telomeres vary. The relationship between telomere length and rheumatic diseases is an ongoing area of research. Most research samples are limited to PBMCs and PBLs, and there is a lack of more detailed studies on telomere length in immune cell populations. Unfortunately, no recent studies directly link telomere shortening in various cell types to rheumatic disease. Though the general understanding is that telomere shortening is observed in various immune cells involved in rheumatic disease, such as T cells and B cells, contributing to the pathogenesis of rheumatic disease, some research also suggests that non-immune cells in affected tissues may exhibit telomere shortening, potentially influencing tissue repair and inflammation. However, they have primarily focused on other autoimmune diseases rather than rheumatic ones. Therefore, we cannot confirm whether telomere shortening is tissue-/organ-specific in the study of rheumatic diseases. For a more comprehensive view, exploring how telomere dynamics might affect other non-immune cell types in affected tissues, such as epithelial cells and synovial fibroblasts, could provide deeper insights. Conclusively, our understanding of telomere length and its role in rheumatic diseases may lead to new treatments that target this feature, enhancing the quality of life for those with these ailments.

## Figures and Tables

**Table 1:** A concise clinical overview of the significance of telomere length in rheumatic diseases.

Disease	Reference	Cells	Method	Authors’ Conclusion
RA	Svyryd et al. [29]	WBC	qPCR	rLTL was significantly shorter in RA patients than at admission. rLTL was shorter in early disease compared to controls. rLTL shortening effects were influenced by age, DET (disease exposure time), and natural rLTL.
	Natalini et al. [104]	PBL	qPCR	Telomere shortening was strongly correlated with RA prevalence but did not lead to the progression of ILD in RA patients.
	Ormseth et al. [101]	PBL	qPCR	RA patients did not exhibit any significant difference in telomere length than healthy controls.
	Prescott et al. [105]	WB	qPCR	Longer prediagnostic LTL was associated with increased RA risk.
	Blinova et al. [108]	PBMC	Q-FISH	Patients with RA had significantly shorter chromosome 4p telomeres.
	Tamayo et al. [109]	PBL	qPCR	TL was significantly longer than controls in RA.
	Fujii et al. [68]	Naive CD4^+^ T cells	TRF	Sluggish cell cycle and growth factor nonresponsiveness.
	Colmegna et al. [107]	CD34^+^ hematopoietic precursor cells (HPCs)	Q-FISH	Marked telomere shortening, sluggish cell cycle progression, and growth factor nonresponsiveness were observed in HPCs from RA patients. This indicates proliferative and oxidative stress-stimulated cell senescence.
	Steer et al. [100]	WBC	TRF	Shortened TRF length in RA patients was not associated with disease markers and severity but was predisposed by the HLA-DRB1 genotype.
	Schonland et al. [99]	PBMC	TRF	Unwarranted loss of telomeres in CD4^+^ T cells was dependent on HLA-DRB1*04 alleles.
	Koetz et al. [106]	PBMC CD4^+^ (45RA/45RO); CD8^+^	TRF	Usage of disease-modifying drugs and disease duration were not related to telomere loss. In contrast, telomere loss was increased in CD4^+^CD45RO^null^ (naive) T cells.
	Yudoh et al. [110]	PBL, synovial infiltrating lymphocytes, and synoviocytes	TRAP	Telomerase activity was elevated in synovial infiltrating lymphocytes and PBL from RA patients, but not in the synoviocytes.
SLE	Bridges et al. [111]	Dried blood spots (DBSs)	qPCR	LTL shortening was accelerated in Black women suffering from childhood SLE.
	Qi et al. [112,113]	PBMC		Increased expression of PINX1 (encoding PinX1 protein, which reduces telomerase enzyme activity and improves telomere length) mRNA in PBMCs.
	Hoffecker et al. [114]	PBMC	qPCR	A higher titer of anti-telomere antibody and shorter telomeres.
	Skamra et al. [115]	WB	qPCR	Telomere shortening in SLE.
	Haque et al. [116]	WB	qPCR	Telomere shortening in SLE.
	Beier et al. [117]	PBMC: T cells (CD4^+^/CD8^+^); B cells (CD19^+^) and monocytes (CD14^+^)	Flow–FISH	SLE did not impact the TL. However, all three lymphocyte subsets exhibit shortened telomeres as compared to monocytes.
	Wu et al. [118]	polymorphonuclear neutrophils mononuclear cells	TRF	SLEDAI augmented telomere erosion.
	Kurosaka et al. [119]	PBMC, T and B lymphocytes	Flow–FISH	TL in T cells from the SLE group was reduced compared to controls but not in B cells.
	Lin et al. [120]	PBMC, T and B lymphocytes	TRAP	B cells from SLE individuals showed shortened TL but did not show differences in TL among T cells.
	Klapper et al. [121]	PBMC, T and B lymphocytes	TRAP	CD19^+^ B cells elevated telomerase activity in SLE patients.
	Kurosaka et al. [122]	PBMC	TRAP	Telomerase activity was significantly correlated with SLEDAI. Younger but not elderly SLE patients had substantially reduced TL.
	Honda et al. [123]	PBMC	TRF	In the early years, CD8^+^ CD4^+^ cells showed increased telomerase, ultimately shortening telomeres.
pSS	Fessler et al. [124]	PBMC	qPCR	Aging signs were increased in naive CD4^+^ T cells.
	Noll et al. [125]	PBMC; saliva; LSG	qPCR	pSS patients showed enhanced telomere erosion in saliva DNA.
	Pringle et al. [126]	SGSC	STELA	SGSCs from samples from patients with pSS were not only lower in number and less able to differentiate but were likely to be senescent.
	Kawashima et al. [127]	lacrimal gland tissue	Q-FISH	pSS patients showed shorter TL, linked with lower p63 and nucleostemin.
SSc	Usategui et al. [128]	PBL	TRF	Shorter age-standardized TL in SSc patients.
	Liu et al. [129]	PBL	qPCR	Dysfunction telomere was linked with SSc-ILD progression.
	Adler et al. [130]	PBL; PBMC	qPCR Flow–FISH	TERF1, an autoantibody against telomere-associated protein, was associated with short TL in lymphocytes and pulmonary fibrosis in patients with SSc.
	Lakota et al. [131]	PBMC	Flow–FISH	Acquired lineage-specific TL shortening in lymphocytes in SSc-associated ILD.
	Tarhan et al. [132]	PBMC	TRAP	Very low telomerase activity in the SSc group.
	MacIntyre et al. [133]	PBMC	TRF	TL was longer in lcSSc individuals, while age-related telomere erosion was not observed but diverged considerably from age-matched healthy individuals only after the age of 50 years.
	Artlett et al. [134]	Lymphocytes; fibroblasts	TRF	Telomeric erosion among SSc patients and family members.
OA	Guillén et al. [135]	PBL	qPCR	Enhanced activity of telomere decay was observed in PBL from OA patients.
	Fajardo et al. [136]	PBL	qPCR	Shortened telomere dynamics in PBL may be a persistent risk sign of knee OA occurrence.
	Mosquera et al. [137]	PBL	qPCR	Telomere size in PBL served as a risk factor for simultaneous knee OA.
	Poonpet et al. [138]	PBL	qPCR	Shortened TL in the knee of OA patients.
	Wiwanitkit et al. [139]	PBL	qPCR	PBL telomere length was associated with prevalent hand OA at baseline
	Zhai et al. [140]	PBL	TRF	Reduced TL in OA individuals.
	Tamayo et al. [109]	PBL	qPCR	No difference in TL was observed among OA patients and age-matched controls.
SpA	Fessler et al. [141]	PBMC	qPCR	CD4^+^ and CD8^+^ T cells subsets showed reduced TL in young SpA patients.
AS	Tamayo et al. [142]	PBL	qPCR	AS patients had longer TL than controls.
AS, PsA	Tamayo et al. [109]	PBL	qPCR	PsA patients showed a higher telomere loss rate than AS patients.
GT	Vazirpanah et al. [143]	PBMC	qPCR	Patients with gout had shorter telomeres.
WG	Vogt et al. [144]	PBMC	TRF	Short telomeres were detected in patients with a disease course of >5 years.

rLTL: relative leukocyte telomere lengths; TL: telomere length; RA: rheumatoid arthritis; SLE: systemic lupus erythematosus; pSS: primary Sjögren’s syndrome; SSc: systemic sclerosis; OA: osteoarthritis; SpA: spondyloarthritis; AS: ankylosing spondylitis; PsA: psoriatic arthritis; GT: gout; WG: Wegener’s granulomatosis; PBL: peripheral blood leukocyte; PBMC: peripheral blood mononuclear cell; WB: whole blood; WBC: white blood cell; LSG: labial salivary gland; SGSC: salivary gland stem cells; qPCR: quantitative polymerase chain reaction; TRF: terminal restriction fragment; Q-FISH quantitative fluorescence in situ hybridization; flow-FISH: flow–fluorescent in situ hybridization; TRAP: telomeric repeat amplification protocol; STELA: single-telomere length analysis; SLEDAI: SLE disease activity index; G-CSF: granulocyte-colony-stimulating factor; HGF: hepatocyte growth factor; VEGF: vascular endothelial growth factor.

## Data Availability

Data sharing is not applicable to this article, as no datasets were generated or analyzed during the current study.
